# Selected papers from the 15th Annual Bio-Ontologies Special Interest Group Meeting

**DOI:** 10.1186/2041-1480-4-S1-I1

**Published:** 2013-04-15

**Authors:** Larisa N Soldatova, Susanna-Assunta Sansone, Michel Dumontier, Nigam H Shah

**Affiliations:** 1 Brunel University, London, UK; 2 University of Oxford, Oxford e-Research Centre, UK; 3 Carleton University, Ottawa, Canada; 4 Stanford University, CA, USA

## Abstract

Over the 15 years, the Bio-Ontologies SIG at ISMB has provided a forum for discussion of the latest and most innovative research in the bio-ontologies development, its applications to biomedicine and more generally the organisation, presentation and dissemination of knowledge in biomedicine and the life sciences. The seven papers and the commentary selected for this supplement span a wide range of topics including: web-based querying over multiple ontologies, integration of data, annotating patent records, NCBO Web services, ontology developments for probabilistic reasoning and for physiological processes, and analysis of the progress of annotation and structural GO changes.

## Summary of selected papers

In 2012, the SIG received 20 paper submissions and 9 flash updates. 15 papers and all the flash updates were selected for presentation at the meeting, out of which 7 papers and 1 commentary appear in this supplement.

The seven papers and the commentary selected for this supplement are extended versions of the original papers presented at the 2012 SIG. The papers include research on web-based querying over multiple ontologies [1,2,7], analysis of the Gene Ontology [3], reports on advances in knowledge representations, e.g. the representation of physiological processes [4] and probabilistic reasoning [6], advances in the annotation of patent records [5], and Web services from the National Center for Biomedical Ontology [8].

The paper titled “Ontology-Based Querying with Bio2RDF’s Linked Open Data” by Callahan et al reports on an update to Bio2RDF [1]. Nineteen new and updated RDF datasets have been mapped to the Semanticscience Integrated Ontology (SIO) to enable federated queries across multiple Bio2RDF endpoints. The new datasets include BioModels—an EBI resource providing details on published computational models primarily from systems biology, BioPortal—a collection of over 300 bio-ontologies from multiple providers, NDC (the National Drug Code Directory)—a Food and Drug Administration (FDA) resource providing a current list of all drugs produced or otherwise processed for distribution by drug companies, and others. Each dataset in the Bio2RDF network is linked to all the other datasets. Federated queries make it possible to formulate a query across connected datasets that reside in separate SPARQL endpoints. Several example SPARQL queries are discussed in the paper and Bio2RDF conversion scripts are available at a GitHub repository http://github.com/bio2rdf/bio2rdf-scripts.

The paper “Biotea: RDFizing PubMed Central in Support for the Paper as an Interface to the Web of Data” by Castro et al demonstrates an approach to the generation of interoperable, interlinked, and self-describing documents in the biomedical domain [2]. The proposed semantic processing approach has been applied to the full-text, open-access subset of PubMed Central. The resulting RDF dataset exploits existing ontologies and semantic enrichment services. The semantic processing of biomedical literature presented in this paper embeds documents within the Web of Data and facilitates the execution of concept-based queries against the entire digital library. The proposed approach delivers a set of tools for metadata declaration and semantic processing of biomedical documents. The model, services, prototype, and datasets are available at http://biotea.idiginfo.org/.

Clarke et al in the paper titled “A task-based approach for Gene Ontology (GO) evaluation” introduce a method for evaluating the GO annotations based on the impact they have on gene set enrichment analysis [3]. The proposed framework uses enrichment analysis to determine the effectiveness of the GO annotations in providing biologically accurate results. As a use case of the evaluation the authors examine how well the GO annotations perform at reproducing biological expectations for a dataset. They demonstrate that the proposed framework enabled the analysis of the progress of annotation and structural GO changes from 2004 to 2012. The authors were also able to determine that the quality of annotations and structure have been improving in terms of their ability to recall underlying biological traits.

The paper titled “Representing physiological processes and their participants with PhysioMaps” by Cook et al presents computable knowledge networks of biological processes and their participants—PhysioMaps [4]. PhysioMaps have originated from the large-scale projects such as the Physiome, the Virtual Physiological Human, and the Virtual Physiological Rat. PhysioMap and SemSim (semantic simulation) models are based on the Ontology of Physics for Biology (OPB). The simulation models are XML files that specify a set of dynamical processes and their participants. Currently the proposed approach supports two types of processes, physical flows and modulation processes. Illustrative examples are provided. The key result is the semi-automatic parsing of biosimulation model code into PhysioMaps that can be displayed and interrogated for qualitative responses to hypothetical perturbations. SemSim project materials are available at http://sbp.bhi.washington.edu/projects/semsim. A tool for creating, annotating, composing and decomposing SemSim models SemGen is available at http://sbp.bhi.washington.edu/projects/semgen.

Eisinger et al in the paper titled “Automated Patent Categorization and Guided Patent Search using IPC as Inspired by MeSH and PubMed” provide a comparative analysis of the Medical Subject Headings ontology (MeSH) and the main patent classification system, the International Patent Classification (IPC) [5]. MeSH supports and improves the document search on PubMed, while patent documents are considerably less accessible. The analysis shows a strong structural similarity of the MeSH and IPC hierarchies, but also some significant differences. The use of IPC to support the patent search comes with two serious disadvantages: complexity of the classification system and sparse class assignments. The low number of IPC class assignments and the lack of occurrences of class labels in patent texts result in the limitations in the patent search. To overcome these limits, the authors propose a system for guided patent search based on the use of class co-occurrence information and the assigning of additional classes to patents.

The paper “Representation of probabilistic scientific knowledge” by Soldatova et al discusses the probabilistic nature of biomedical knowledge and the necessity for an ontological support for probabilistic reasoning with scientific knowledge [6]. The authors propose an ontology HELO (HypothEses and Laws Ontology) to model the key entities of the theory of probability. HELO is designed to consistently accommodate scientific hypotheses and laws collected from different sources: interviews with scientists, web pages, research papers, databases, program codes. The authors demonstrate the utility of HELO on three worked examples: changes in the probability of the hypothesis that sirtuins regulate human life span; changes in the probability of hypotheses about gene functions in the *S. cerevisiae* aromatic amino acid pathway; and the use of active learning in drug design, where a strategy for the selection of compounds with the highest probability of improving on the best known compound was used. HELO is available at https://github.com/larisa-soldatova/HELO.

Vita et al in the commentary “Query enhancement through the practical application of ontology: the IEDB and OBI” outline their experiences in utilizing bio-medical ontologies to provide enhanced database search functionality [7]. The authors analyse the benefits of the information captured by a formal ontology implemented directly into the user web interface for querying databases. The authors discuss the long-term goal of enabling a true semantic integration of data and knowledge in the biomedical domain. Vita et al describe their progress towards this goal and the main obstacles. The discussed approach is considered on the example of the Immune Epitope Database (IEDB, http://www.iedb.org) that utilizes the Ontology for Biomedical Investigations (OBI) and several additional ontologies to represent immune epitope mapping experiments.

Whetzel on behalf of the NCBO Team in the review paper “NCBO Technology: Powering semantically aware applications” provides an overview of technology developed by the National Center for Biomedical Ontology (NCBO), a National Center for Biomedical Computing created under the NIH Roadmap [8]. The NCBO developes Web services, which provide access to one of the largest repositories of biomedical ontologies. This overview describes typical services provided by NCBO for the research community. For example, the Ontology Web services provide access to ontologies, navigation of the class hierarchy and details of each term and the NCBO Annotator Web service “tags” text automatically with terms from ontologies in BioPortal. The NCBO Widgets package enables the Ontology Web services to be used directly in web sites. The functionality of the NCBO Web services and widgets are incorporated into semantically aware applications for ontology development and visualization, data annotation, and data integration. The NCBO Web services are documented at: http://www.bioontology.org/wiki/index.php/NCBO_REST_services.

## Competing interests

The authors have no competing interests to declare.

## Declarations

This article has been published as part of *Journal of Biomedical Semantics* Volume 4 Supplement 1, 2013: Proceedings of the Bio-Ontologies Special Interest Group 2012. The full contents of the supplement are available online at http://www.jbiomedsem.com/supplements/4/S1
